# Cost Effectiveness of a Home-Based Intervention That Helps Functionally Vulnerable Older Adults Age in Place at Home

**DOI:** 10.1155/2012/680265

**Published:** 2011-08-16

**Authors:** Eric Jutkowitz, Laura N. Gitlin, Laura T. Pizzi, Edward Lee, Marie P. Dennis

**Affiliations:** ^1^Jefferson School of Population Health, Doris N. Grandon Center for Health Economics and Outcomes Research, 1015 Walnut Street, Suite 319, Philadelphia, PA 19107, USA; ^2^The Aging Intervention Center at Johns Hopkins, 525 Wolfe Street, Suite 316, Baltimore, MD 21205, USA; ^3^Jefferson School of Pharmacy, 130 South 9th Street, Suite 1540, Philadelphia, PA 19107, USA; ^4^Jefferson Center for Applied Research on Aging and Health, Thomas Jefferson University, 130 South 9th Street, Suite 500, Philadelphia, PA 19130, USA

## Abstract

Evaluating cost effectiveness of interventions for aging in place is essential for adoption in service settings. We present the cost effectiveness of Advancing Better Living for Elders (ABLE), previously shown in a randomized trial to reduce functional difficulties and mortality in 319 community-dwelling elders. ABLE involved occupational and physical therapy sessions and home modifications to address client-identified functional difficulties, performance goals, and home safety. Incremental cost-effectiveness ratio (ICER), expressed as additional cost to bring about one additional year of life, was calculated. Two models were then developed to account for potential cost differences in implementing ABLE. Probabilistic sensitivity analyses were conducted to account for variations in model parameters. By two years, there were 30 deaths (9: ABLE; 21: control). Additional costs for 1 additional year of life was $13,179 for Model 1 and $14,800 for Model 2. Investment in ABLE may be worthwhile depending on society's willingness to pay.

## 1. Introduction

The primary health problems confronting older adults are chronic and can affect their ability to carry out everyday self-care [1]. Functional difficulties significantly compromise quality of life and are associated with increased frailty, depression, nursing home placement, and mortality [2, 3]. Numerous interventions have been tested in randomized trials that may help older adults with late-life disability age in place at home [4, 5]. Nevertheless, research shows that functionally vulnerable elders continue to receive inadequate care [6–9]. To reverse this situation and enhance wide-scale adoption and implementation of proven programs in service settings, economic evaluations of promising interventions must be conducted [10, 11]. 

Only a few home-based interventions targeting older adults with late-life disability have been evaluated for cost effectiveness with studies demonstrating cost savings. However, most of these studies have involved European programs or preventive approaches not specifically designed to improve function or reduce mortality in at-risk vulnerable older adults living at home in the USA [12–14]. 

One promising home intervention tested in the USA is Advancing Better Living for Elders (ABLE) [15, 16]. ABLE was previously tested in a two-group randomized parallel trial with 319 older adults who had difficulties with instrumental or daily activities of living. ABLE was designed to address client-identified functional difficulties, performance challenges at home, and home safety concerns. Compared to a no-treatment control group, ABLE was shown to reduce functional difficulties and enhance home safety and self-efficacy to manage daily functional challenges at 6 and 12 months. Moreover, ABLE reduced mortality at 12 and 24 months from study entry [15–18].

Given ABLE's promising outcomes and to extend an understanding of the benefits of this intervention, we conducted an economic analysis post hoc. The purpose of this study is to estimate the cost effectiveness of implementing ABLE from the perspective of a homecare agency. Only costs associated with the implementation of ABLE were considered and two cost scenarios were developed to account for potential cost differences in implementing ABLE. These analyses used an incremental cost-effectiveness ratio (ICER) with the primary outcome measure of life years saved (LYS) over two years.

## 2. Methods

### 2.1. Study Design and Sample

The original ABLE trial was conducted between 2000 and 2003 with survivorship followed out to December 2005. As previously reported [15], trial participants were 70 years or older, cognitively intact, and living at home with functional difficulties. Participants were recruited through service agencies and media announcements. Of the 319 participants enrolled, 159 were randomized to ABLE and 160 to a no-treatment (usual care) control group. For this study, baseline interview data was used to characterize the sample. Data from the National Death Index (NDI) records were used to determine length of time of survivorship up to December 31, 2005.

### 2.2. Intervention

ABLE participants received five occupational therapy (OT) contacts (four 1.5-hour visits and one brief telephone contact) and one 1.5-hour physical therapy (PT) home visit over the first six months. OTs identified and prioritized functional difficulties, and provided strategies to modify the environment, enhance safety, and minimize performance difficulties. OTs identified home modification needs and, with client approval, coordinated product ordering, delivery, and implementation through the Housing Department of the Philadelphia Corporation for Aging (the region's area agency on aging). PTs provided balance and muscle strengthening exercises, fall recovery techniques, and referral for additional therapy if necessary. In the following six months (maintenance phase), participants received three brief OT telephone calls to reinforce strategy use. A final OT home visit provided closure. 

Control group participants did not receive intervention contact. At study completion (12-month interview), participants received a home safety booklet free to the public.

### 2.3. Cost-Effectiveness Model

A decision analytic model was constructed for the cost-effectiveness analyses using TreeAge Pro 2009 statistical software. Two models were constructed to account for variation in cost estimates. Model 1 (base case) reports on estimated costs of delivering ABLE in a home care agency. Model 2 (base case + 10%) accounts for a potential variation in the cost of delivering ABLE in a real world setting.

### 2.4. Cost

Costs were calculated based on recommendations of the US Public Health Service Panel on Cost-Effectiveness and major peer-reviewed journals [1, 19–23]. All costs inputs are reported in Table 2; costs were captured in 2003 dollars to reflect when the original data was collected and then adjusted to 2010 dollars. Costs of the intervention reflected five direct categories; OT/PT home and telephone sessions, staff training, intervention materials, therapist travel, and home modifications (ordering, installing, and quality control). 

 Time spent by OTs delivering the intervention was estimated to be 1.5 hours per home visit and 15 minutes per telephone call. This estimate was derived from reviewing the study design and post hoc interviews with OT study interventionists. Per-hour pay for OTs was calculated using national average rates ($28) for OTs with one to four years experience [24], with an additional 25% added to account for fringe benefits. Also, 15 minutes were estimated to account for preparation and documentation for each session. 

 We estimated the time spent by the PT conducting the intervention (1.5 hr/home visit) based on a review of the study design and post hoc interviews with interventionists. Per-hour pay for the PT was calculated using the national average rate of $31 for PTs with one to four years experience; an additional 25% was assumed for fringe benefits [24]. An additional 15 minutes were estimated to account for preparation and documentation following the session based on therapist records. 

Staff training time for seven OTs and one PT was 16 hours and involved instruction in the study protocol. Similar assumptions for hourly wage rates as above were applied. Cost of training was estimated on a per-participant basis. To estimate cost on a per-participant basis we calculated the total cost of training and divided this number by the number of study participants in the intervention group.

There were two types of material costs: (1) those used by interventionists ($5) and (2) education print materials provided to participants ($10). Total cost of materials per participant was estimated to be $15. 

Interventionist travel expenses to and from participant homes were calculated based on an average of a 20 mile radius round trip per visit, reimbursed at the government rate of $.51 a mile [25]. 

 Home modification (e.g., grab bars and raised toilet seats) costs included ordering, purchasing, installing, and assuring quality.

### 2.5. Outcome Measure—Life Years Saved (LYS)

For the cost-effectiveness analysis, we used LYS over two years as the primary outcome measure. The survival benefit of ABLE compared to control has been described elsewhere [18]. Briefly, to determine survival benefit, the number of days to death was calculated from the baseline interview until date of death or December 31, 2005 using data from the National Death Index. Kaplan-Meier method was used to analyze survival rate at two years from date of study entry [18]. Difference in area under the Kaplan-Meier curve was then used to estimate LYS.

### 2.6. Discounting

Because the effects of the ABLE intervention occurred over a period of two years, it is necessary to account for the time delay of the benefit as it is more advantageous to receive a benefit earlier rather than later [21, 22]. To adjust for the time delay of a benefit, we discounted our outcome measure, life years saved, by a factor of 3%. Costs were not discounted because they were incurred only during the first year of the study.

### 2.7. Incremental Cost-Effectiveness Ratio (ICER)

The ICER was calculated by taking the difference in cost between the intervention and the control group divided by the difference in survival benefit between treatment and control groups [22, 23]. The ICER therefore represents the additional costs to bring about one life year saved from the intervention compared to usual care.

### 2.8. Sensitivity Analyses

In order to account for uncertainties in our model, probabilistic sensitivity analyses (PSA) were performed on both Models 1 and 2. To conduct a PSA, each variable in the model is assigned a mean and distribution around its mean. TreeAge Pro 2009 was used to calculate the PSA. To derive the results of the analysis, the mean incremental cost and effect, TreeAge Pro 2009 runs 1000 microsimulations. During each simulation, the computer uses the distribution around each variable to generate average costs and effects. Based on the average costs and effects over 1000 microsimulations, the computer then estimates the mean incremental cost and effect. Results from the PSA are presented as an acceptability curve. The acceptability curve graphically illustrates the probability of the intervention being cost effective over a range of willingness-to-pay values. 

To be consistent with the methodology of the PSA, each variable in Models 1 and 2 was assigned a distribution of values based on the standard deviations calculated during the initial study. However, for some variables (e.g., occupational therapist time on phone), data was not uniformly available from the clinical trial. For these cases, we derived estimations from consulting with research staff or the literature.

## 3. Results

### 3.1. Study Participants

Characteristics of the study population have been presented elsewhere [15]. Briefly, there were no large or statistically significant differences between intervention and control group participants at baseline on demographic and health variables (Table 1).

### 3.2. Cost

Total cost of ABLE per participant was $942 (Table 2). Cost for the no-treatment control group was $0 given that no program treatment was received. In Model 2 (base case + 10%), cost of ABLE was $1,036.

### 3.3. Outcome Measure—Life Years Saved

By two years, 30 study participants had died; 9 deaths in the intervention and 21 deaths in the control group. Based on previously published Kaplan-Meier survival analysis [18], the intervention group (*n* = 160) had a survival rate of 94% (*n* = 9 deaths) reflecting a mortality rate of 6%; this is in comparison to the control group (*n* = 159) which had a survival rate of 87% or a mortality rate of 13% (*n* = 21 deaths; *P* =  .02). The difference between mortality rates represents the additional survival benefit of ABLE.

### 3.4. ICER and Sensitivity Analysis of ICER Estimate

Under the assumptions of Model 1, the ICER (cost per one additional year of life) was $13,179 and under the assumptions of Model 2, the ICER is $14,800. 

**Figure 1:**
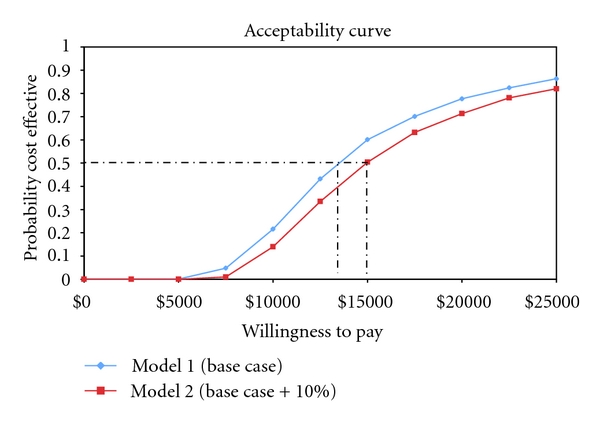


Figure 1 details the acceptability curve for Models 1 and 2. Based on the acceptability curve and under the assumptions of Model 1, ABLE is cost effective greater than 50% of the time as long as a purchaser is willing to pay more than $13,000 for one additional year of life. Under the assumptions of Model 2, ABLE is cost effective greater then 50% of the time as long as the purchaser is willing to pay more than $14,800 for one additional year of life.

## 4. Discussion

To our knowledge, this is one of the first cost-effectiveness analyses of a home-based intervention tested in the USA which reduced functional difficulties and mortality risk in vulnerable older adults. The original ABLE trial did not include cost as a study aim and thus the cost analyses presented here were post hoc and hence necessarily exploratory. Our study demonstrates, however, the value of conducting cost analyses even post hoc to derive preliminary economic conditions of effectiveness and enhance the implementation potential of existing proven programs for vulnerable older adults. There are two key findings from this study. First, ABLE's cost effectiveness is within an acceptable range of willingness to pay (WTP) values identified in previous related studies, although research is very limited in this area. Second, the cost of ABLE is reasonable and compares favorably to other nonpharmacologic, home-based interventions for older adults. 

Traditionally, cost-effectiveness analyses use quality-adjusted life years (QALYs) as the primary outcome measure and apply a WTP threshold of $50,000 per quality-adjusted life (QALY). However, in ABLE, QALYs were not captured in the original trial, a potential limitation of this economic study. Thus, we were unable to use this standard metric to evaluate cost effectiveness. To aid in the interpretation of our findings and compensate for the lack of QALY outcomes, we searched the literature for studies which evaluated WTP for interventions that decrease morality. By searching for established WTP values, we sought to determine the value of one additional year of life, as reported in the literature. Applying WTP values published previously to ABLE provides a preliminary contextual basis for understanding our derived ICER estimates. 

Our search yielded only two studies that can provide some insight as to WTP for ABLE [26, 27]. Johannesson and Johansson [26] estimated the WTP ($400–$1500) for a one-year increase in life expectancy of a hypothetical intervention. Taking the average of this range ($950) and adjusting for inflation, we arrived at an estimated WTP of $1,299/year. Applying Johannesson and Johansson WTP estimate to the acceptability curve generated in our study, ABLE would not be cost effective under either Model 1 or Model 2 [26]. 

However, Johannesson and Johansson WTP estimate was low compared to other published studies [26, 28]. One possible explanation for the low estimate is that Johannesson and Johansson surveyed a Swedish population and, thus, their preferences may not be the same as a US population. In addition, the population surveyed was younger (<69) than the ABLE population (mean age 79). 

In another study, Johnson et al. evaluated WTP by asking respondents how much they would be WTP for one additional year of life based on six quality of life scenarios (no physical limitations and no social limitations, some physical limitations and no social limitations, some physical limitations and some social limitations, home bound, need help, and in hospital) [27]. The authors found that individuals were willing to pay the most for the scenario in which they had no physical or social limitations. The ABLE population could be described as having some physical and social limitations [15, 16]. Johnson et al. found the WTP interval (after adjusting for inflation and currency exchange rates) for this subgroup to be between $1,754 and 17,556. If we were to apply the upper range (>$14,800) of the Johnson et al. WTP estimates to our acceptability curve (Figure 1), ABLE would be considered cost effective greater than 50% of the time. 

Although the WTP data that is applicable to ABLE is limited, these two studies provide some basis for contextualizing the ABLE ICER estimate. While it is difficult to make generalizations about the cost effectiveness of ABLE given limited WTP data, utilizing the acceptability curve (Figure 1), an individual decision maker can determine the probability of ABLE being cost effective given their own WTP. More importantly, the dearth of WTP data indicates the need for future studies to collect and report on such values for older adults with late-life disability. 

The cost of implementing ABLE relative to similar programs is also difficult to evaluate as there are limited studies on the cost of similar novel home-based interventions. An OT program for well elderly reported average program costs of $548 per participant with cost per QALY for the intervention estimated at $10,666 [12]. While average costs for ABLE were $400 higher, the difference is chiefly due to costs associated with specialized equipment ($439) important to vulnerable elders or those aging at home with functional difficulties. 

ABLE also compares favorably to an OT dementia caregiver intervention tested in The Netherlands [13]. Intervention costs per patient in The Netherlands study were $1,738 (USD), and the intervention was found to be successful only 36% of the time. 

Finally, ABLE compares favorably to The Geriatric Resources for Assessment and Care of Elders model (GRACE) [11]. GRACE is 2-year home based care management intervention designed to improve quality of care and reduce acute care. The mean cost of GRACE per patient per year was $1,000 [29], almost identical to ABLE. However, unlike in ABLE, there was no statistical difference in mortality rate between the intervention and control arms of the GRACE study.

As this is a post hoc study, we were unable to derive real-time costs, a study limitation. Our method for estimating the cost of delivering ABLE was based on a review of the study protocol and interviews with intervention staff. Although we were thorough in our analyses, we believe that our cost estimate may in fact overestimate the cost of ABLE because it does not take into account potential cost savings. For example, those in the ABLE group benefited from a decrease in functional difficulties and mortality. Thus, there is a strong possibility that those in the ABLE group compared to the control group actually used less health care services. Unfortunately, the original data does not lend itself to an estimate of health care utilization, a significant limitation.

Several other study limitations should also be noted. First, we were unable to conduct our analyses from a societal perspective and, thus, some may view this as major study limitation. Secondly, traditional cost-effectiveness analyses use QALYs as the primary outcome measure instead of QALYs, we used life years saved. Although it would have been ideal to include QALYs in this analysis, as stated above, the original parent trial did not capture this data. 

In conclusion, although there are limitations to all cost-effectiveness analyses, these studies are at the forefront of a growing trend in health economics to quantify benefits of proven programs from which to make judgments as to what should be translated into real-world services. With the aging of the population, it is increasingly important to measure cost effectiveness of programs that help older adults remain independent in their homes. To advance services and policies that support aging in place, economic analyses of promising programs are important. Few existing proven programs for functionally vulnerable older adults have included cost analyses prospectively. However, we show in this study that it is possible to evaluate the cost of a proven program post hoc, although admittedly there are limitations to a retrospective approach and it is preferable to conduct such analyses prospectively. Nevertheless, our approach offers a preliminary understanding of the costs of a highly effective program. The cost of ABLE can be considered low in view of the high cost of medical and drug therapies. Also, the results from the cost-effectiveness analyses of ABLE show that the additional cost to bring about one additional year of life to older adults living at home with functional difficulties compares somewhat favorably to the very few studies conducted in this area. Future studies of ABLE and other related programs will need to be conducted in which treatment effects are measured over a longer period of time and cost analyses are considered a priori to study implementation so as to capture cost from a societal perspective. Finally, future studies that use nontraditional outcome measures need to place ICER estimates in real world context by evaluating an individual and a society's willingness to pay for such benefits.

##  Conflict of Interests

The authors declare that there is no conflict of interests.

##  Authors' Contribution

Jutkowitz conceptualized the paper and cost analyses, conducted all cost and sensitivity analyses, and had primary responsibility for preparation of the paper. L. N. Gitlin, Ph.D., was principal investigator of the original randomized ABLE trial. She provided guidance as to the description of the intervention and associated cost categories and helped to develop and edit the introduction and discussion sections of the paper. L. T. Pizzi, Pharm. D., provided consultation on appropriate cost analyses and helped to refine the presentation of the cost data. E. Lee provided consultation on appropriate cost analyses and conducted the sensitivity analyses. M. Dennis, Ph. D., helped prepare the data set for which analyses were conducted and reviewed and edited the paper for accuracy.

## Figures and Tables

**Table 1:** Background characteristics.

Characteristic	Control (*n* =159)	Experimental (*n* = 160)	Total (*N* = 319)	*P*
Mean Age (SD)	78.5 (5.7)	79.5 (6.1)	79.0 (5.9)	.158
Race (%)				.387
White	52.2	53.1	52.7	
African	45.9	45.0	45.5	
American				
Hispanic	0.0	1.3	0.6	
Other	1.9	0.6	1.2	
Gender (%)				.751
Male	18.9	17.5	18.2	
Female	81.1	82.5	81.8	
Living arrangement (%)				.462
Alone	59.7	63.8	61.8	
With others	40.3	36.3	38.2	
Education (%)				.916
< High school	37.7	35.6	36.7	
High school	30.2	31.9	31.0	
> High school	32.1	32.5	32.3	
Mean number of health conditions (SD)	7.1 (2.8)	6.7 (2.7)	6.9 (2.7)	.295
MMSE	27.0 (1.8)	26.8 (1.8)	26.9 (1.8)	.346

MMSE: Mini-mental status examination.

**Table 2:** Cost categories for ABLE program.

Cost categories	Cost (range)
(1) Time spent with ABLE participants	
OT preparation	$57 ($44–$67)
OT contact	$299 ($239–$359)
PT preparation	$10 ($8–$12)
PT contact	$58 ($46–$69)
(2) Training	
OT/PT	$5 ($4–$6)
(3) Materials	
For OT/PT	$5 ($4–$6)
For participants	$10 ($8–$12)
(4) Travel	
Mileage	$61 ($49–$73)
(5) Home modifications	
Modifications	$439 ($351–$527)
Total average cost per person	$942
